# Serotype distribution and antimicrobial resistance of *Streptococcus pneumoniae* in children with acute bacterial meningitis in Mozambique: implications for a national immunization strategy

**DOI:** 10.1186/s12866-016-0747-y

**Published:** 2016-06-29

**Authors:** Aquino Albino Nhantumbo, Eduardo Samo Gudo, Juliana Caierão, Alcides Moniz Munguambe, Charlotte Elizabeth Comé, Tomás Francisco Zimba, Milton Ozório Moraes, Cícero Dias, Vlademir Vicente Cantarelli

**Affiliations:** Laboratório Nacional de Referência de Microbiologia, Instituto Nacional de Saúde, Ministério da Saúde, Maputo, Mozambique; Universidade Feevale, Rio Sul, Brazil; Universidade Federal de Ciências de Saúde de Porto Algre (UFCSPA), Porto Alegre, Brazil; Departamento de Medicina at the Hospital Central de Maputo, Maputo, Mozambique; Fundação Oswaldo Cruz, Rio de Janeiro, Brazil; Instituto Nacional de Saúde, Ministério da Saúde, Maputo, Mozambique; Laboratório Nacional de Referência de Microbiologia, Instituto Nacional de Saúde, Av Eduardo Mondlane 1008, PO Box 264, Maputo, Mozambique

**Keywords:** Pneumococcal meningitis, Mozambique

## Abstract

**Background:**

*S. pneumoniae* is the leading cause of acute bacterial meningitis (ABM) in children. Vaccination using the 10-valent conjugate vaccine (PCV-10) was recently introduced into the National Immunization Program in Mozambique, but data on serotype coverage of this vaccine formulation are scarce. In this study, we investigated the serotype distribution and antimicrobial resistance of isolates of *S. pneumoniae* causing ABM in children < 5 years at the two largest hospitals in Mozambique.

**Methods:**

Between March 2013 and March 2014, a total of 352 cerebrospinal fluid (CSF) samples were collected from eligible children, of which 119 (33.8 %) were positive for *S. pneumoniae.* Of these, only 50 samples met the criteria for serotyping and were subsequently serotyped using sequential multiplex PCR (SM-PCR), but 15 samples were non-typable.

**Results:**

The most common serotypes of *S. pneumoniae* were 1 (18.2 %), 5 (15.2 %), 14 (12.1 %), 9 V (12.1 %), 23 F (9.1 %), 6A (9.1 %), 4 (9.1 %) and 6B (6.1 %). Serotypes 1, 5, 9 V, 6A and 12 were mostly prevalent in Northern Mozambique, while serotypes 23 F, 4, 6B, 3 and 15B were predominant in Southern. Serotype coverage of PCV-10 and PCV-13 vaccine formulations were 81.8 % and 93.9 %, respectively. Serotypes 1, 3, 4, 6B, 14, 23 F were resistant to penicillin and sensitive to ceftriaxone.

**Conclusions:**

Our findings shows that changing the current in use PCV-10 vaccine formulation to PCV-13 formulation might increase substantially the protection against invasive strains of *S. pneumoniae* as the PCV-10 vaccine formulation does not cover the serotypes 3 and 6A, which are prevalent in Mozambique.

## Background

*Streptococcus pneumoniae* is the leading cause of otitis media, sinusitis, bacteremia, pneumonia, meningitis and sepsis in children. Worldwide, an estimated 1.6 million of children under the age of 5 die every year as a consequence of *S. pneumoniae* related diseases [1–3]. The elderly and immunosuppressed individuals are also vulnerable [4].

Meningitis is a serious consequence of invasive disease caused by *S. pneumoniae,* leading to more than 100,000 deaths annually in children under the age of 5 [2, 3]. Recent estimates demonstrate that up to 90 % of these deaths occurs in resource-limited settings, mostly in sub-Saharan Africa and Asia [3, 5].

Pneumococcus is an antigenically complex bacteria and currently, more than 95 serotypes have been described [6–8]. These serotypes differ in term of pathogenesis, target organ, age, geographical distribution, virulence and susceptibility to antibiotics [9].

Globally, the most common serotypes are 1, 5, 14, 6A, 6B, 19 F and 23 F, of which serotypes 1, 5, 6A and 14 occur predominantly in developing countries, whereas serotypes 19 F, 6B, 23 F and 14 occur mostly in developed countries [10, 11]. In recent years, the rapid spread of antimicrobial resistant *S. pneumoniae* strains and the emergence of multidrug-resistant strains (MDRSP), has increased the global concern for pneumococcal infection [12].

In regard to Mozambique, data on serotype distribution of *S. pneumoniae* are scarce, and with the exception of one study conducted in Manhiça district, a rural area in southern Mozambique [13], no other study has yet been conducted. Therefore, data from Manhiça district has few limitations, such as, they are more than 10 years old and, taken into consideration that Manhiça is a rural village, these data are difficult to generalize to the entire country given the size and geographic diversity of Mozambique, particularly in terms of climate, demographic, socio-economic and cultural aspects [14]. In this regard, it is possible that serotype distribution and antimicrobial sensitivity of *S. Pneumoniae* may vary in different regions of the country. This is particularly important in a time in which Mozambique recently introduced the PCV-10 vaccine formulation into the Extended Program of Immunization.

In this context, this study was conducted with the purpose of determining the frequency and distribution of capsular serotypes of *S. pneumoniae* causing acute bacterial meningitis in children under 5 years of age at the two largest hospitals in the country, Maputo Central Hospital, in the Southern region, and Nampula Central Hospital, in the Northern region of the country. This study will generate information on the serotype coverage of pneumococcal conjugate vaccine formulations and present baseline data for future assessments of the impact of pneumococcal vaccination in Mozambique.

## Methods

### Study design, study sites and target population

This study was implemented as part of the routine sentinel surveillance system for pediatric acute bacterial meningitis (ABM), implemented by the National Institute of Health (INS) in Mozambique. The sentinel surveillance system for ABM was established in March 2013 at two regional hospitals, Maputo Central Hospital (HCM) and Nampula Central Hospital (HCN). Both are regional referral hospitals and offer several specialized medical services, including inpatient and outpatient care across all age groups.

The HCN is situated in Nampula province, the most populous in the country, has 23 districts with a total of 3,985,613 inhabitants [14]. The paediatric ward at HCN has 184 beds. The HCM is situated in Maputo city, the capital of Mozambique, with a total of 1,766,823 inhabitants [14]. HCM is the largest hospital in the country, with 322 beds in the paediatric ward. All children admitted to these health facilities with clinical presentation of ABM between March 2013 and March 2014 were eligible for enrolment.

### Case definition

Per WHO guidelines, suspected case of ABM is defined as a child aged < 5 years with sudden onset of fever (>38.5 °C rectal or 38.0 °C axillary) and at least 1 of the following signs: neck stiffness or flaccid neck, bulging fontanel, convulsion, irritability, or drowsiness.

### Samples collection and questionnaire

Upon admission, lumbar puncture (LP) was aseptically performed by a trained pediatrician on each child who met the case definition for ABM.

A standard case investigation form was used to collect clinical and demographic data.

### Laboratory testing

#### Bacteriological testing of CSF samples at sentinel site

As part of routine surveillance for ABM, cerebrospinal fluid (CSF) samples were initially cultured at the sentinel sites onto 5 % sheep blood agar plates (MAST, Merseyside, UK) and all growing isolates were stored between 2-8 °C and shipped to the National Reference Microbiology Laboratory (NRML) in Maputo for further testing.

#### Identification of *S. pneumoniae* by culture at NRML

At the NRML, all isolates received from the sentinel site were frozen at -80 °C until further testing. Isolates were recovered by plating onto 5 % sheep blood and chocolate agar plates (MAST, Merseyside, UK). Twenty-four hours after incubation at 35 ° C ±2 ° C with 5 % of CO_2_, bacterial isolates were identified by colony morphologic analysis and growth requirement. Pneumococci were identified based on morphological features in Gram stain, optochin susceptibility test (OXOID – DD1 OPTOCHIN, Basingstoke, England) and bile solubility (BD – BBL Desoxycholate Reagent Droppers, Becton Dickinson and Company, USA) [15]. All isolates of *S. pneumoniae*, were used for molecular serotyping.

#### DNA extraction from CSF samples and *S. pneumoniae* isolates

Two hundred microliters of all CSF samples and isolates of *S. pneumoniae* previously identified by culture were used for DNA extraction. DNA was extracted using either Biopour mini kit (BIOPUR, Biometrix diagnostic, Brazil), or High pure PCR template kit (Roche Diagnostics Corporation, US), according to the manufacturer’s instructions. Total DNA was eluted in 200 μl of appropriated elution buffer and stored at −20 °C until use.

#### Diagnosis of *S. pneumoniae* using PCR

Molecular diagnosis of *S. pneumoniae* was performed as part of the routine diagnosis of meningitis within the national surveillance system for pediatric ABM, implemented by the National Institute of Health. Routinely, the molecular diagnosis of meningitis is performed using M-qPCR. This assay was based on SYBER detection system (Platinum SYBR Green qPCR Supermix-UDG, Invitrogen, California, UK) using the LightCycler 2.0 instrument (Roche Diagnostic GmbH, Mannheim, Germany). This detects simultaneously *Neisseria meningitidis*, *Streptococcus pneumoniae,* and *Haemophilus influenzae* using a set of specific primers as described by Ke *et al*., 2000 [16]; Corless *et al*., 2001[17] and El Aila *et al*., 2011 [18].

#### Serotyping of *S. pneumoniae*

Serotyping was performed on all isolates of *S. pneumoniae* and all CSF samples from PCR positive children, whose cycle threshold (ct) value was lower than 30. CSF samples from PCR positive children whose ct value was higher than 30, were excluded, due to insufficient DNA for serotyping. Serotyping was performed using the Sequential multiplex PCR (SM-PCR), adapted for regional algorithm, and covering the 39 most common serotypes involved in the etiology of invasive pneumococcal diseases (IPD) in Africa, and grouped into 8 multiplex PCR reactions [19]. The capsular polysaccharide (*cps*) was used as internal control. The reaction was performed in a final volume of 25 μl. Each reaction mix contained buffer solution at 2x, 0.2 mM concentration of each deoxynucleoside triphosphate (dNTPs), reaction buffer 10x (100 mM of Tris-HCl, pH 8.5 and 500 mM of KCl), 1.7 mM of MgCl_2,_ 1.5U⁄ μl of Taq DNA polymerase, primers at the concentration from 0.1 to 0.5 uM and 5 μl of template DNA. The PCR assays were performed in a thermocycler (GeneAmp PCR System 9700, PE Applied Biosystems). Samples were amplified as follows: an initial denaturation step at 95 °C for 15 min, followed by 35 cycles at 95 °C for 30 seconds (denaturation), 54 °C for 90 seconds (annealing) and 72 °C for 1 min (elongation). After amplification, PCR products were analyzed by gel electrophoresis.

#### Antimicrobial susceptibility test

Minimum inhibitory concentrations (MICs) to penicillin, erythromycin, vancomycin, trimethoprim-sulfamethoxazole, tetracycline and ceftriaxone were determined using E-test® (Biomereux, SA) strips*.* The results were interpreted according to Clinical and Laboratory Standards Institute guidelines (CLSI, 2014) [20]. The reference strain *S. pneumoniae* ATCC 49619 was used as the quality control.

### Statistical analysis

Data were entered into a database developed using Epi Info version 3.5.4 (CDC U.S.A.) and was analyzed using SPSS statistical software version 20 (IBM, U.S.A.). Categorical variables were reported as proportion and statistical significance differences were assessed using Pearson Chi-square test with a significance level of less than 0.05.

## Results

### General characteristics of study participants

Between March 2013 and March 2014, a total of 352 CSF samples were collected from children clinically suspected of ABM who were admitted to two referral hospitals. The median age of study participants was 9 months (IQR 4 - 30 months) and 52.8 % (186/352) of them were male. More than half of the children were aged less than 12 months, while children of the age group 12-23 months and 24-59 months comprised 11 % (39/352) and 31 % (109/352) of the study group (Table 1). Children from Nampula Central Hospital, represented 73.6 % from the sample (*n* = 259), while children from Maputo Central Hospital represented 26.4 % (*n* = 93). Children from HCM and HCN were similar in term of their age (median: 17.4 months for HCN and 20.3 months for HCN, respectively) and gender (male gender = 51.6 % in HCM and 53.3 % in HCN, respectively). *S. pneumoniae* was identified in a total of 119 (33.8 %) samples, of which 17 were initially identified by culture and the remaining 102 were identified by PCR. Serotyping for *S. pneumoniae* using SM-PCR was performed in a total of 50 samples, comprising the 17 isolates of *S. pneumoniae* identified by culture and 33 PCR positive CSF samples for which their ct value was inferior to 30. Most of children with positive result for *S. pneumoniae* and children with typable CSF sample were aged below 12 months, following the same pattern of enrolled children (Table 1).

**Table 1:** Age and gender distribution of enrolled patients, patients with positive and negative CSF results and patients with typable CSF samples

Characteristics	Total (*n* = 352)	*S. pneumoniae* positive (*n* = 119)	Children with typable CSF (*n* = 33)
Age			
0-11 months	204 (58.0 %)	77 (64.7 %)	24 (72.7 %)
12-23 months	39 (11.0 %)	18 (15.1 %)	3 (9.1 %)
24-59 months	109 (31.0 %)	24 (20.2 %)	6 (18.2 %)
Gender			
Male	186 (52.8 %)	66 (55.5 %)	13 (39.4 %)
Female	166 (47.2 %)	53 (44.5 %)	20 (60.6 %)

### Serotype distribution and coverage rate of pneumococcal vaccine formulations

**Fig. 1:**
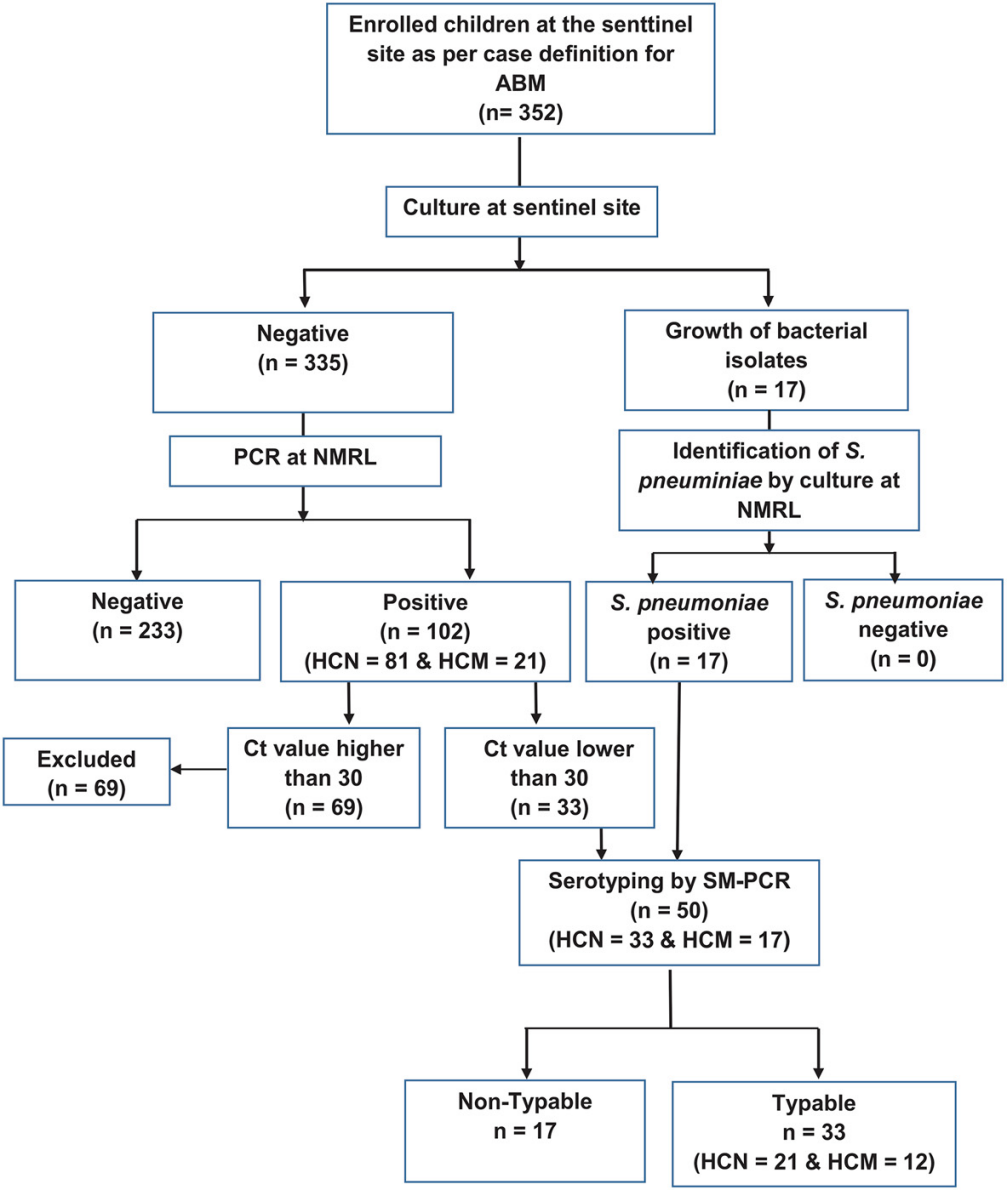
Flowchart of sample collection and testing. The flow chart depicts the number of CSF samples collected from of children under 5 years old at each sentinel site and tested at sentinel site and NMRL between March 2013 and study March 2014. Abbreviations. CSF: Cerebrospinal fluid; HCM: Hospital Central de Maputo; HCN: Hospital Central de Nampula; SM-PCR: sequential multiplex polymerase chain reaction; NMRL: National Reference Microbiology Laboratory

Of the 50 samples for which serotyping was performed, a total of 33 (66.0 %) were typable and their corresponding serotype was identified, the remaining 17 samples were not-typeable (SM-PCR NT), as shown in Fig. 1. The most frequent serotypes were 1 (18.2 %, 6/33), 5 (15.2 %, 5/33), 14 (12.1 %, 4/33), 9 V (12.1 %, 4/33), 23 F (9.1 %, 3/33), 6A (9.1 %, 3/33), 4 (9.1 %, 3/33), 6B (6.1 %, 2/33), 12 (3.0 %, 1/33), 15B (3 %, 1/33) and 3 (3.0 %, 1/33). Serotype distribution of children older than 2 years old was significantly different of that of children younger than 2 years old (Chi-Square = 28; *p* = 0.001).

**Fig. 2:**
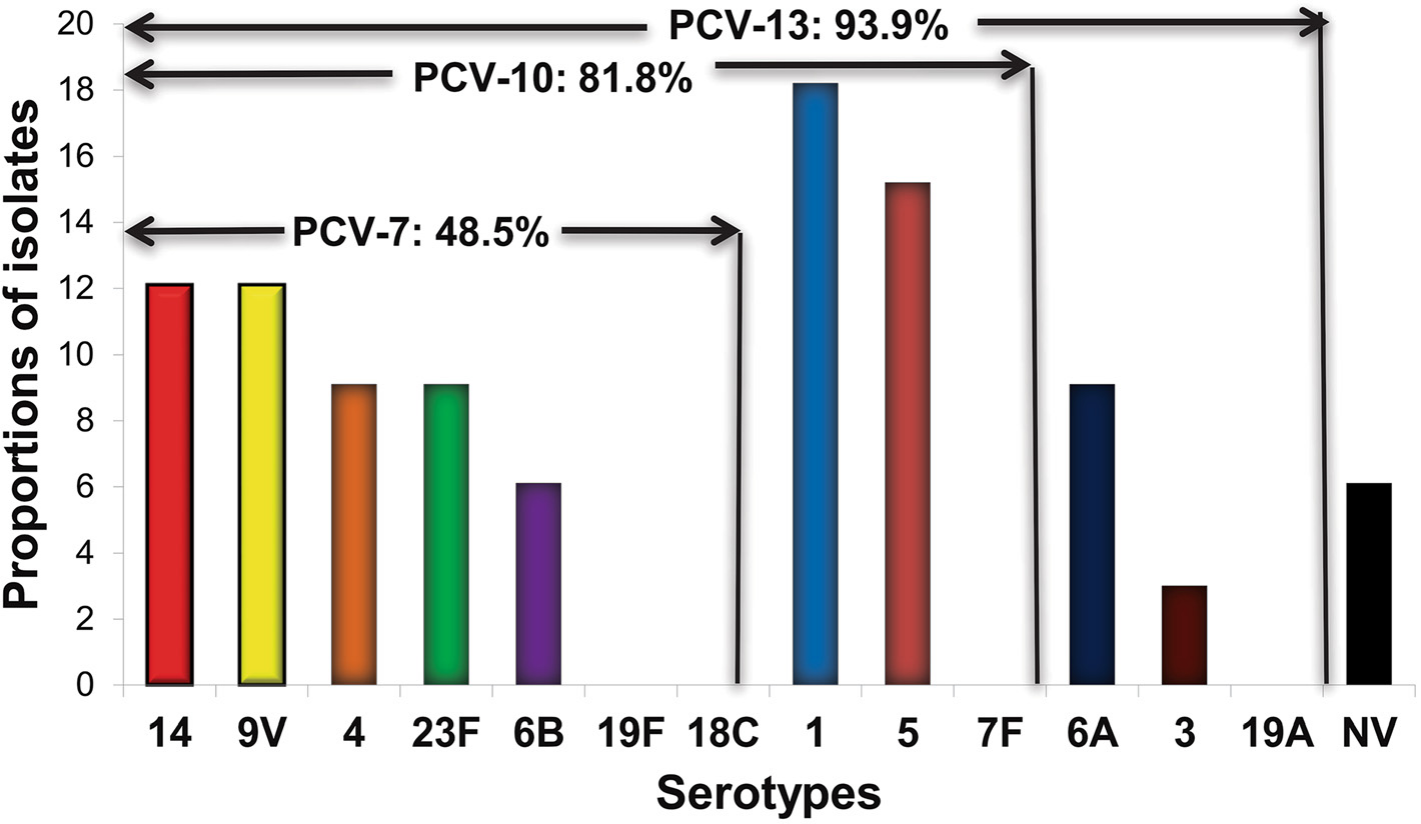
Distribution of serotypes of *S. pneumoniae* and vaccine coverage rates for PCV-7, PCV-10 and PCV-13 vaccine formulations. Each bar represents the relative frequency of each serotype of *S. pneumoniae*. The value in the arrows above the bars depicts the vaccine coverage rates for PCV-7, PCV-10 and PCV-13, respectively NV, serotypes not included in 13-valent pneumococcal conjugate vaccine. NV, nonvaccine serotypes (serotypes 12 and 15B)

Figure 2 shows that the rate of vaccine coverage against the serotypes of *S. pneumoniae* causing pediatric meningitis in Mozambique was 48.5 % (16/33), 81.8 % (27/33), and 93.9 % (31/33) for PCV-7, PCV-10, and PCV-13, respectively.

**Fig. 3:**
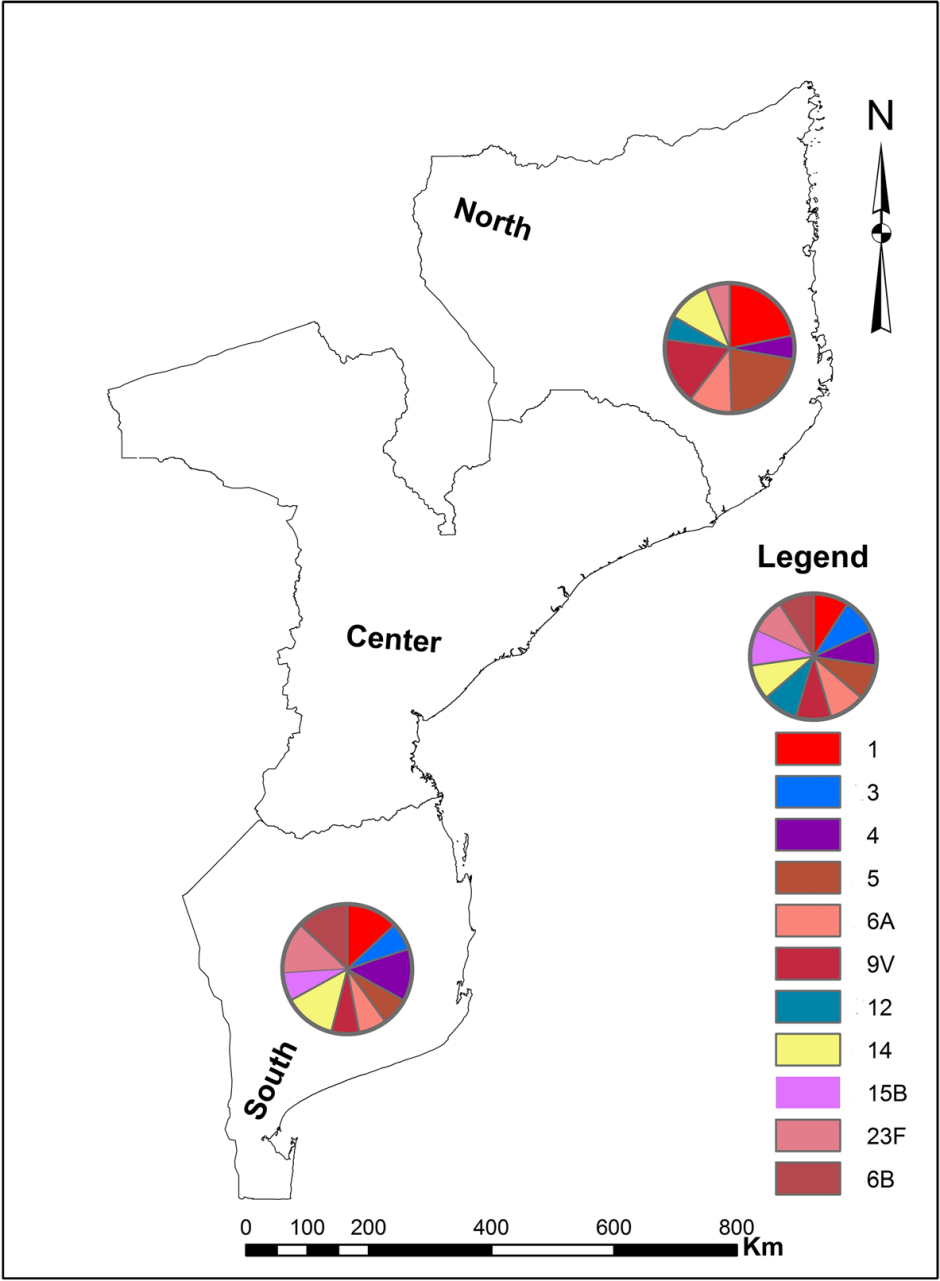
Distribution of serotypes of *S. pneumoniae* stratified by region, between March 2013 and March 2014. Figure depicts the distribution of *S. pneumoniae* serotypes in the southern (HCM) and northern (HCN). Each color represents one serotype

The serotypes 1, 5, 9 V, 6A and 12 were mostly found in Northern Mozambique, while serotypes 23 F, 4, 6B, 3 and 15B were reported mostly in the Southern Mozambique (Fig. 3). In addition, the non-PCV-10 serotype 3 and 15B (frequency of 3.0 % for both isolates) were found in Maputo city, situated in southern Mozambique, while the non-PCVs serotype 12 (frequency of 3.0 %) was found in Nampula city, situated in northern part of the country.

### Antimicrobial susceptibility profile of isolates of *S. pneumoniae*

Table 2 summarises the antibiotic susceptibility profile of pneumococcal serotypes, and showed that 88.2 % of serotypes were resistant to penicillin (MIC > 0. 125 μg/mL), according to the revised CLSI breakpoints for parenteral penicillin (resistant ≥ 0.12 mg/mL for meningitis isolates), while for ceftriaxone all serotypes were susceptible (100 %). The susceptibility rate for erythromycin, vancomycin, chloramphenicol and tetracycline were 76.5 %, 88.2 % and 64.7 %, 35.3 %, respectively.

**Table 2:** Antibiotic susceptibility profile of *S. pneumoniae* serotypes

*Serotype*	No. of isolates	MIC (μg/ml) of^a^						
		PEN (n/N)	ERY (n/N)	SXT (n/N)	VANC (n/N)	CRO (n/N)	TCY (n/N)	CHL (n/N)
		R^a^	R	R	R	R	R	R
14	4	4/4	1/4	4/4	0/4	0/4	3/4	0/4
6A	2	2/2	0/2	2/2	2/2	0/2	2/2	2/2
4	2	2/2	1/2	2/2	0/2	0/2	1/1	1/2
3	1	1/1	0/1	1/1	0/1	0/1	1/1	0/1
1	1	1/1	0/1	1/1	0/1	0/1	0/1	0/1
15B	1	0/1	0/1	1/1	0/1	0/1	0/1	1/1
23 F	2	2/2	2/2	2/2	0/2	0/2	2/2	1/1
9 V	1	0/1	0/1	1/1	0/1	0/1	0/1	0/1
NT	3	3/3	0/3	3/3	0/3	0/3	2/3	1/3
Total	17	15/17	4/17	17/17	2/17	0/17	11/17	6/17

PEN, penicillin; ERY, erythromycin, SXT, trimethoprim-sulfamethoxazole; VANC, vancomycin; CRO, ceftriaxone; TCY, tetracycline; CHL, chloramphenicol. NT, not typeable

^a^Resistant

## Discussion

In this study we described for the first time the serotype distribution of *S pneumoniae* at the two largest hospitals in Mozambique, and provided baseline data for further assessments of the impact of pneumococcal vaccination.

Findings from our study show that in Mozambique, the predominant serotypes of *S. pneumoniae* causing ABM in children aged <5 year were 1, 5, 14, 9 V, 23 F, 6A and 4. This finding is similar to a recent study conducted in South Africa [21], but is different than that reported in studies conducted in Nigeria [22] Burkina Faso and Togo, which found that serotypes 14, 1 and 18 were the most prevalent [23].

Of note, our data suggests that the distribution *S. pneumoniae* serotypes in the Northern differs of that in the Southern, as shown in the Fig. 3. However, these findings should be interpreted with caution because of the small size of our sample, which is not representative of the entire population in the country. Studies with larger size should be conducted to confirm these results.

In regard to serotype coverage of PCV-10, data from our study show that this vaccine formulation covers 81.8 % (27/33) of *S. pneumoniae* strains identified in this study. Similar results were found in a previous study conducted in Manhiça District, a rural community situated in southern Mozambique [13], South Africa [21], and other sub-Saharan countries situated in the African meningitis belt, such as Burkina Faso, Togo [24, 25] and Nigeria [22]. Our data show that PCV-13 vaccine formulation would increase the serotype coverage from the current 81.8 %, to 93.9 % because PCV-13 formulation provide added protection against the serotypes 3, 6A and 19A.

Our results also show that serotype 1 was the most common cause of pneumococcal meningitis in Mozambique, which is in agreement with findings from other Sub-Saharan Africa countries [13, 22, 24]. The high prevalence of serotype 1 might be a consequence of a previous epidemics caused by this serotype in Mozambique, as has been reported in other settings, such as Ghana [10].

Additionally, data from our study show that serotype 3 occurs in children, although with low frequency. This is relevant because serotype 3 is an important cause of invasive disease in children [9] and has been associated with high fatality rates [26, 27].

Serotypes 12 and 15B, known as non-vaccine serotypes (NV) occurred in 6 % of the children. This is an important finding, because these serotypes are not included in the existing PCVs vaccine formulations. Similar findings were reported in studies conducted in South Africa and other sub-Saharan Africa countries [21, 28].

In regards to antimicrobial susceptibility profile, resistance of *S. pneumoniae* to penicillin was high, but this result cannot be generalized due to the small number of isolates tested in this study. This finding is in agreement with previous studies, which also suggest that the rate of resistance of *S. pneumoniae* to penicillin and other antibiotic classes is increasing [29–34]. Resistance to penicillin was also observed for serotypes 1, 3, 4, 6B, 14, 23 F. These results are similar to those reported in recent studies conducted in South Africa [21], Algeria [33] and China [35].

An important finding of this study was the fact that two *S. pneumoniae* strains belonging to serotype 6A presented a decreased susceptibility to vancomycin (MIC = 1.52 μg/mL, for both). Our results corroborate finding from a recent study conducted by *Ataee et al*, 2014 in Iran, which reported emergence of vancomycin tolerant, strains of *S. pneumoniae.* In his report, *Ataee et al*, 2014 found that 8 % (4/46) of *S. pneumoniae* strains presented a lower susceptibility to vancomycin as measured by assessment of MIC [36]. In fact, the number of studies reporting the emergence of meningitis caused by *S. pneumoniae* strains that are tolerant to vancomycin is increasing [37, 38]. These patients have been shown to have a poorer survival rate than patients with meningitis caused by nontolerant strains [37, 38]. Another study conducted by *Sanaei et al* found that among *S. pneumoniae* carrier’s children, the proportion of *S. pneumoniae strains* resistant to vancomicin was 1.5 % [39]. Altogether, this represents an important warning that vancomycin resistant or tolerant strains of *S. pneumoniae* are emerging as a serious public health threats.

We found that 17 out of 50 isolates were not-typeable. The reasons why these samples were not-typeable are unknown, however, molecular serotyping as it’s considered the gold standard method for serotyping. Moreover, multiplex PCR has been shown to be more accurate in determining the serotype of *S. penumoniae* as compared to serological methods [22, 40]. In term of sensitivity of this method, our sequential multiplex PCR (SM-PCR), was adapted for regional algorithm, and covers the 39 most common serotypes involved in the etiology of invasive pneumococcal diseases (IPD) in Africa. We argue that emerging isolates in Africa belonging to some other serotypes, not covered by the 39 serotypes scheme may be present and would need further consideration.

To our knowledge, this is the first description of *S. pneumonieae* serotype distribution in Northern Mozambique.

We would like to acknowledge some limitations of our study, which may influence the interpretation of the results and extrapolation to the entire population. For example, the relatively small size of our sample, in particular from Maputo Central Hospital might have influenced our results and findings, in particular, the number of strains used for determining the antibiotic susceptibility rate was very small. In addition, the information was incomplete in several of the standard case investigation forms such as vaccination status.

## Conclusion

Our study provides preliminary findings on serotype distribution of *S. pneumoniae* causing ABM in Southern and Northern Mozambique showing that the serotype coverage of the recently introduced PCV-10 vaccine formulation against the serotypes of *S. pneumoniae* circulating in Mozambique is 81.8 %, as opposed to the coverage of 93.9 % if we were using PCV-13 vaccine formulation, suggesting that changing from the currently in use PCV-10 formulation to PCV-13 can save additional lives. Lower coverage of PCV-10 is attributed to the emergence of non-PCV-10 serotypes 3 and 6A.

## Abbreviations

ABM, acute bacterial meningitis; ATCC, american type culture collection; CLSI, laboratory standards institute guidelines; CSF, cerebrospinal fluid; ct, cycle threshold; DNA, deoxyribonucleic acid; dNTP, deoxynucleoside triphosphate; HCN, nampula central hospital; INS, National Institute of Health; IPD, invasive pneumococcal diseases; IQR, interquartile range; LP, lumbar puncture; MDRSP, multidrug-resistant strains; MIC, minimum inhibitory concentration; M-qPCR, multiplex qualitative polymerase chain reaction; NRML, national reference microbiology laboratory; PCV-10, ten-valent conjugate vaccine; PCV-13, thirteen -valent conjugate vaccine; SM-PCR NT, not-typeable by sequential multiplex polymerase chain reaction; SM-PCR, sequential multiplex polymerase chain reaction; UDG, uracil DNA glycosylase; UFCSPA, Universidade Federal de Ciências de Saúde de Porto Alegre
